# Human lymphoid tissue sampling for vaccinology

**DOI:** 10.3389/fimmu.2022.1045529

**Published:** 2022-11-17

**Authors:** Karolina M. Kwiatkowska, Catherine G. Mkindi, Carolyn M. Nielsen

**Affiliations:** ^1^ Department of Biochemistry, University of Oxford, Oxford, United Kingdom; ^2^ Department of Intervention and Clinical Trials, Ifakara Health Institute, Bagamoyo, Tanzania

**Keywords:** bone marrow, lymph node, fine needle aspirate (FNA), B cells, vaccine, Tfh cells, germinal centre (GC), long-lived plasma cells (LLPCs)

## Abstract

Long-lived plasma cells (LLPCs) – largely resident in the bone marrow – secrete antibody over months and years, thus maintaining serum antibody concentrations relevant for vaccine-mediated immunity. Little is known regarding factors that can modulate the induction of human LLPC responses in draining lymph node germinal centres, or those that maintain LLPCs in bone marrow niches following vaccination. Here, we review human and non-human primate vaccination studies which incorporate draining lymph node and/or bone marrow aspirate sampling. We emphasise the key contributions these samples can make to improve our understanding of LLPC immunology and guide rational vaccine development. Specifically, we highlight findings related to the impact of vaccine dosing regimens, adjuvant/vaccine platform selection, duration of germinal centre reactions in draining lymph nodes and relevance for timing of tissue sampling, and heterogeneity in bone marrow plasma cell populations. Much of this work has come from recent studies with SARS-CoV-2 vaccine candidates or, with respect to the non-human primate work, HIV vaccine development.

## Introduction

Vaccines are among public health’s most effective tools for combatting infectious disease but a poor understanding of the underlying immunological mechanisms frequently impedes vaccine development. One of the greatest perennial issues for the vaccinology field is a lack of knowledge of how to induce *durable* immune responses in the target populations. While many vaccine candidates generate encouraging peak antibody concentrations, these often wane rapidly in the following weeks or months. If circulating antibody is required for protection, this rapid decay can be highly concerning.

**Figure 1:**
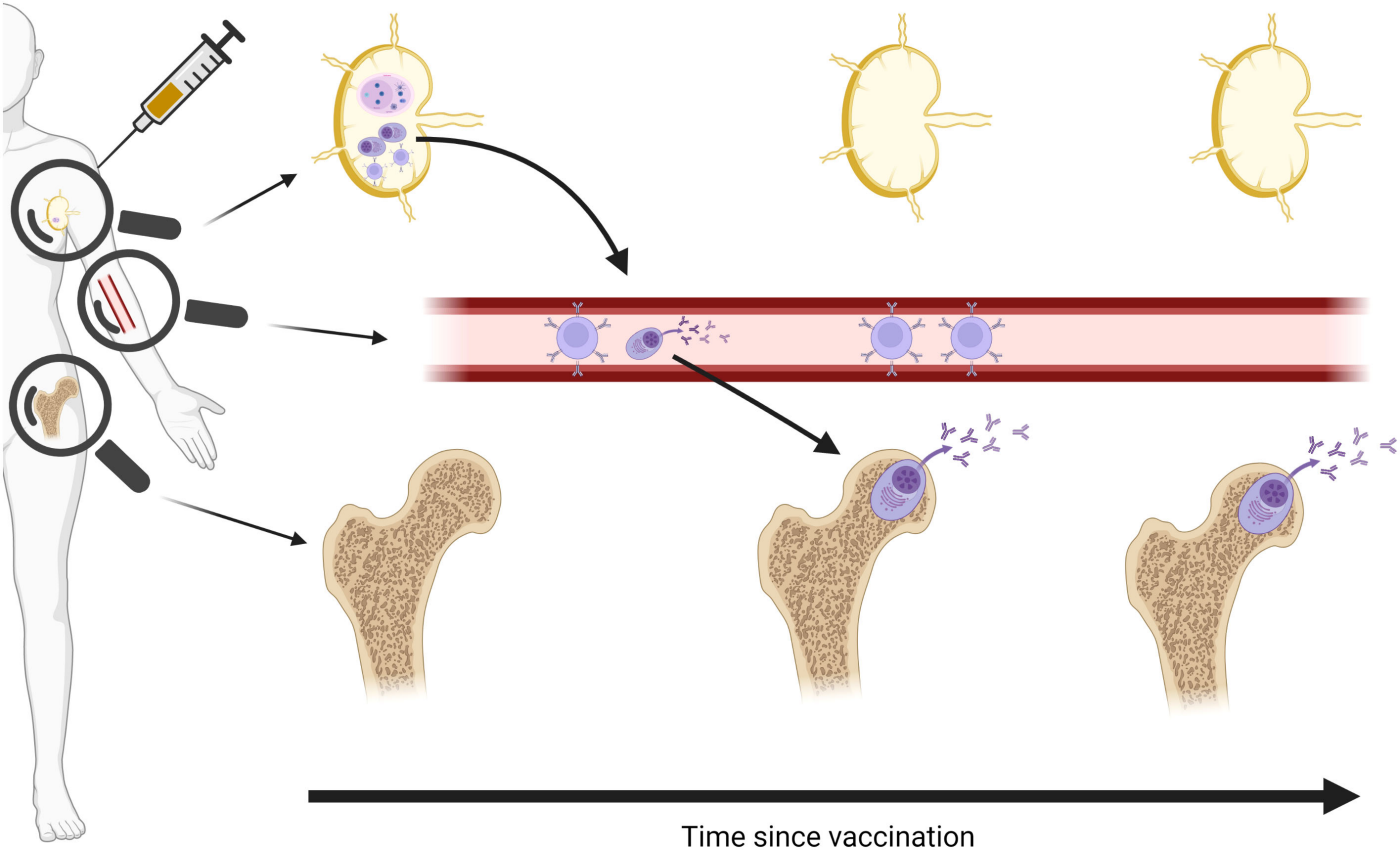
Schematic of vaccine-specific B cell responses following vaccination. Vaccine antigen traffics to draining lymph node and induces a germinal centre (GC) response to generate memory B cells and long-lived plasma cell (LLPC) precursors. Memory B cells remain in circulation, waning over time, while LLPCs are seeded in the bone marrow and remain stable over months and years. Magnifying glasses indicate different immune cell populations detectable in different compartments. Created in BioRender.

Lymphoid tissue-resident long-lived plasma cells (LLPCs) are the only cells that continuously produce antibody over months and years, and are therefore responsible for the longevity of the humoral response. Two particularly central, but poorly understood, processes are the generation of the LLPC-precursors in draining lymph node germinal centres (GCs) following vaccination, and subsequent migration to the bone marrow where they steadily secrete antibody and thus maintain humoral immunity over time (**Figure 1**). As human vaccine immunology has historically relied almost exclusively on analyses of circulating blood to characterise immune responses, opportunities to interrogate GCs and bone marrow niches have been missed.

Long-term serology data demonstrates that immunogens do not all induce equivalent LLPC responses (1, 2). Unfortunately, information about the factors that influence generation of vaccine-specific LLPCs remains scarce as too few human vaccine clinical trials have incorporated the necessary lymphoid tissue sampling. There is some evidence of putative LLPC-precursors in the blood (3), but as yet no published data linking these cells to bone marrow LLPCs in the context of vaccination. In the absence of clear understanding on how modifiable vaccination parameters – such as platform/adjuvant/dosing – modulate induction of vaccine-specific LLPC in draining lymph node GCs and subsequent bone marrow seeding, it is difficult for vaccine developers to target a durable LLPC response.

In this review, we highlight the rare human and non-human primate vaccination studies that have incorporated draining lymph node and/or bone marrow aspirate sampling and the major findings yielded from these samples (**Table 1**). Where quantitative values are clearly stated in publication text or tables, these are included here. Methodologies to analyse vaccine-specific B cells, in various compartments – including CyTOF, CITE-seq, LIBRA-seq, Beacon and other cutting-edge approaches – have been reviewed elsewhere (15–18). An overview of vaccine-specific responses in other lymphoid tissues – e.g. GCs in tonsils (19, 20) or plasma cells in the gut (21) or spleen (6) – are also outside the scope of this review.

**Table 1:** Human vaccine studies including draining lymph node or bone marrow aspirate antigen-specific immunogenicity analyses.

Publication(chronological order)	Antigen/Pathogen	Vaccine Platform	# doses	Draining lymph node	Bone marrow
Mei et al., 2015. *Blood* (4)	Tetanus	N/A; historical vaccination; observational study		*	X
Halliley et al., 2015. *Immunity* (5)	Influenza, tetanus, measles, mumps				X
Groves et al., 2018. *Blood Adv* (6)	Diphtheria/tetanus/pertussis, influenza, polio				X
Eberhardt et al., 2020. *J Virol* (7)	Varicella Zoster virus	live attenuated virus	1-2		X
Turner et al., 2020. *Nature* (8)	Influenza	inactivated virus	1	X	
Davis et al., 2020. *Science* (9)	Influenza	inactivated virus	1		X
Turner et al., 2021. *Nature* (10)	Spike (SARS-CoV-2)	mRNA (BNT162b2)	2	X	
Mudd et al., 2021. *Cell* (11)	Spike (SARS-CoV-2)	mRNA (BNT162b2)	2	X	
Law et al., 2022. *iScience* (12)	Influenza	inactivated virus	1	X	
Lederer et al., 2022. *Cell* (13)	Spike (SARS-CoV-2)	mRNA (BNT162b2)	2	X	
Kim et al., 2022. *Nature* (14)	Spike (SARS-CoV-2)	mRNA (BNT162b2)	2	X	X

*Lymph node samples in Mei et al. not analysed for antigen specificity.

X, sample taken and analyses performed to detect vaccine-specific responses. Includes clinical trials or observational studies where vaccine-specific cellular analyses were performed with lymph node and/or bone marrow aspirate samples.

## Draining lymph nodes

GC reactions have profound importance for the generation of protective vaccine-specific memory B cells and LLPCs following vaccination. While the memory B cells output can be quantified by assaying peripheral blood, many features of GC responses remain obscure in the absence of lymph node sampling: GC B cells are only detectable in secondary lymphoid organs like lymph nodes. In particular, data is limited on the duration of GC reactions, which may have great relevance for affinity maturation of BCRs and the development of broadly neutralising antibodies (bnAbs). Recapitulating the lymph node microenvironment *in vitro* has historically been difficult, but a recently published GC organoid system shows great potential for future interrogation of lymph node GC activity using lymph node samples (22). Efforts to develop a lymphoid follicle “organ-on-a-ship” may similarly prove a useful tool (23). GC organoids generated from draining lymph node aspirates could be used to model a variety of key facets of humoral immunity including GC longevity, affinity maturation, class-switch recombination, B-Tfh cell interactions, and memory B cell output.

Draining lymph node sampling is feasible through either lymph node biopsy or fine needle aspirates (FNAs) under ultrasound guidance. Early work in the context of vaccination comes from the oncology field where lymph node sampling has been long-established for diagnostic purposes. In the vaccination context, this work includes analyses of lymph node biopsies from melanoma patients for detection of vaccine-specific T cells following a gp100_209-2M_ peptide vaccine co-administered with montanide (24, 25). A comparison of these two approaches – including processes of volunteer consent and follow-up – has been reviewed previously in the context of lymph node sampling for HIV vaccine development (26). Studies in non-human primates have further demonstrated that FNA and biopsy samples correlate well in terms of GC Tfh and B cell quantitation (27). However, it should be noted that for studies where spatial analysis of lymph node architecture is of interest, the biopsy approach would be more appropriate (e.g (28). Another limitation of FNA sampling is the relatively low number of cells obtained (**Table 2**), though certainly sufficient for flow cytometry or single-cell RNA-sequencing.

**Table 2:** Average cell yields from healthy adult draining lymph node fine needle aspirates.

Vaccination study?	Publication	# cells/FNA (average)	# cells/FNA (range)	# serial FNAs/volunteer
Yes	Ben-Othman et al. 2020. *Front Immunol* (29)	*Average and range not reported* 8/31 samples > 3.0 x 10^4^ cells		2
Yes	Turner et al., 2020. *Nature* (8).	8.9 x 10^5^	2.3 x 10^4^ – 6.9 x 10^6^	4
Yes	Turner et al., 2021. *Nature* (10)	*Not reported.*		5
Yes	Mudd et al., 2021. *Cell* (11)	1.7 x 10^5^ (CD4+ T cells)^	1.5 x 10^3^ – 5.5 x 10^5^ (CD4+ T cells)^	6
Yes	Law et al., 2022. *iScience* (12)	1.1 x 10^6^ (lymphocytes)	6.8 x 10^3^ – 1.2 x 10^7^ (lymphocytes)	2
Yes	Lederer et al., 2022. *Cell* (13)	*Not reported.*	3.0 x 10^5^ – 4.0 x 10^7^	2
Yes	Kim et al., 2022. *Nature* (14)	*Not reported.*		6^¬^
No	Tatovic et al., 2015. *J Immunol* (30)	3.3 x 10^6^ (leucocytes)	1.0 x 10^6^ – 1.0 x 10^7^	2
No	Yang et al., 2019. *Front Immunol* (31)	7.2 x 10^5^	8.0 x 10^4^ – 1.6 x 10^6^	1
No	Havenar-Daughton et al., 2020. *J Immunol Methods* (32)	2.2 x 10^5^ *	4.0 x 10^3^ * – 7.9 x 10^6^ *	1 (2 sites)*

^Yield data from Mudd et al. relates to CD4+ T cell counts as acquired by flow cytometry.

*Yield data from Havenar-Daughton et al. relates to median values from axillary lymph node samples.

^¬^In 6/15 volunteers in Kim et al., a second draining lymph node was also identified and sampled.

Here, we will focus on the FNA approach due to several clear advantages over biopsy for vaccination studies a) feasibility of longitudinal (repeat) sampling, b) reduced theoretical disturbance to immune response, and c) less invasive procedure with minimal recovery time. Multiple groups have published the methodology for human (29–32) or non-human primate (NHP) (33) FNA sampling but use in human vaccine clinical trials or observational studies for detection of vaccine-specific GCs still remains rare (8, 10, 13, 14). Identification of the correct (draining) lymph node is clearly key to relevant analyses; in human vaccination studies this is the axillary site following intramuscular vaccination in the deltoid. Ultrasound guidance by a skilled radiologist is required to detect the lymph node of interest.

Seminal work by Turner and colleagues – in the first report of vaccine-specific GC B cells in humans – used serial draining lymph node aspirate sampling by FNA to track influenza haemagglutinin (HA)-specific GC B cell clones following seasonal vaccination (*n*=8) (8). Here, the authors were able to address the question of whether seasonal influenza vaccination induced plasma cells derived from GC B cell responses in addition to those derived from peripheral memory B cell differentiation. FNAs were taken at baseline and then 1-, 2-, 4-, and 9-weeks following a single vaccination; 7/8 vaccinees yielded FNAs with sufficient cells for analysis. HA-specific plasmablasts (HA^+^CD38^+^CD20^lo^) and/or activated B cells (HA^+^CD38^-^CD20^+^) were detected in FNAs from all volunteers by flow cytometry using biotinylated recombinant HA at a minimum of 1 post-vaccination time point. HA-specific cells were detected within the GC B cell (IgD^lo^CD38^int^CD20^hi^) compartment in 5/7 volunteers, peaking at 2-weeks post vaccination (range: 0.00-83.65%) and persisting out to 9-weeks post vaccination in 3/7 vaccinees [range: 0.00-25.37%; Extended Data from (8)]. It is unclear if the remaining 4 vaccinees completely lacked GC responses to vaccination. Detecting and evaluating such heterogeneity in human GC responses post-vaccination may be highly informative for understanding variation in vaccine-mediated efficacy across a range of pathogens. Indeed, the authors speculate based on these immunokinetics and clonality analyses that the early polyclonal antibody response in first 4 weeks is likely derived from the early plasmablast (short-lived plasma cell) response, while later antibody comes from GC-derived LLPCs.

Later, working with samples from SARS-CoV-2 mRNA BNT162b2 vaccinees, Turner, Mudd and colleagues conducted two studies focused on understanding spike protein-specific GC responses in draining lymph nodes (10, 11). In the first study, FNAs from draining lymph nodes were taken before the second dose and then 1, 2, 4 and 12 weeks after the booster (*n*=14). Spike-specific GCs were detected using fluorophore-conjugated spike probes in all vaccinees at the time of the booster (3-weeks after the first dose) followed by heterogeneous transient increases. Unlike the influenza study, the peak response was unclear; frequencies of spike-specific GC B cells (CD10^+^CD3^-^IgD^lo^BCL6^+^CD38^int^) within the CD19+ population were generally increased until at least 4 weeks following booster (*n*=11; range: 0.04-15.37%), with 8/10 vaccinees still near peak 12-weeks after the booster [*n*=10; range: 0.02-20.12%; Extended Data (10)]. These responses appear to be an improvement over the aforementioned seasonal influenza vaccination study where HA-specific GCs were detected only in 3/7 vaccinees by 9-weeks, though a potential role for differences in historical antigen exposure was not explored (8). Given this discrepancy in influenza/SARS-Cov-2 GC responses, the authors suggest a significant role for the mRNA-lipid nanoparticle platform in improving antigen dissemination and adjuvanting, at least as compared to an inactivated whole virus vaccine platform.

The potential insights from lymph node analysis are not limited to B cells. In the second study, Mudd and colleagues reported Tfh cell responses in the same cohort of BNT162b2 vaccinees with FNAs at Day 0, 21, 28, 35, 60, 110 and 200 [*n*=15 (11)]. Here, the authors detected interesting differences in spike-specific Tfh cells in lymph nodes versus circulating in peripheral blood. Tetramers for an immunodominant epitope were used to monitor kinetics of vaccine-specific CD4^+^ T cells in HLA-DPB1*04-positive vaccinees. While peripheral spike-specific Tfh cells peaked a week after the booster vaccination (median: 0.01%; range: 0.00-0.15% of total CD4^+^ T cells) and then contracted to undetectable levels, spike-specific Tfh cells in lymph node FNAs persisted at high frequencies with 2/5 vaccinees assayed still at high frequencies at 6-months (Day 200). The total lymph node Tfh population furthermore correlated with spike-specific GC B cells at matched time points (*n*=95; Days 21 to Day 200).

Law and colleagues also investigated GC Tfh responses – in this case following seasonal influenza vaccination – detecting an increase in CD38^+^ICOS^+^ GC Tfh in draining lymph nodes 5-days following vaccination as compared to baseline and correlation of this subset with GC B cells [*n*=16 (12)]. These early indicators of GC activation were restricted to the draining lymph nodes; an increase in CD38^+^ICOS^+^ Tfh cells was not observed in contralateral lymph nodes and no increase was observed in activated Tfh in peripheral blood (circulating Tfh) despite the emergence of antigen-specific CD4^+^ T cells by this time point. The authors propose this 5-day time point is too early for emergence of antigen-specific or activated Tfh into the periphery from lymph node GCs.

Focusing on a comparison between GC responses in healthy (*n*=15) and immunocompromised (kidney transplant; *n*=14) adult BNT162b2 vaccinees comes a study from Lederer and colleagues (13). An increase in spike- and RBD-specific GC B cells was detectable in ipsilateral draining lymph nodes in healthy vaccinees between prime (RBD-specific within GC B cells, median [range]: 0.02% [0.00-0.19%]) and booster vaccinations (0.05% [0.00-1.17%]). No baseline FNAs were obtained. In contrast, the GC B cell population was entirely absent from immunocompromised vaccinees; no spike- or RBD-specific GC B cells were detectable in the draining lymph nodes. The B cell response was not completely abrogated, however, as mBC responses were observed in the draining lymph node and peripheral blood albeit at lower frequencies to healthy vaccinees. This data provides useful insights into mRNA vaccine immunogenicity in the context of immunocompromise. Indeed, without lymph node analyses the severity of the impact on GC output would have been missed.

Finally, a report by Kim and colleagues with mRNA BNT162b2 vaccinees directed at understanding the maturation dynamics of the spike-specific GC responses and integrating with analyses of bone marrow LLPCs (discussed further below) was the first human vaccination study to incorporate analyses of both key lymphoid tissues (14). FNAs were taken on a similar schedule as above with the addition of an extra time point 26-weeks after booster vaccination. Spike-specific GC B cells were detectable in 10/15 vaccinees at this late time point, showing two-thirds of vaccinees maintained spike-specific GC reactions for at least 6 months. Furthermore, affinity maturation appears to continue during this time with a 2.5-fold increase detected in somatic hypermutation (SHM; i.e. nucleotide mismatches from germline sequence) in spike-specific GC B cells between 1-week and 12-weeks after booster vaccination. Spike-specific lymph node plasma cells were also detected with similar kinetics, though at lower frequencies, and this flow cytometry data was supported by clustering of single-cell RNA sequencing of lymph node samples where 33.8% of lymph node B cells were identified as GC B cells versus 7% as plasma cells. Analyses of the two populations indicated high clonal overlap and rate of somatic hypermutation suggesting draining lymph node plasma cells differentiate from draining lymph node GC B cells. There was also a varying extent of clonal overlap between spike-specific GC B cells and peripheral blood plasmablasts 1-week after booster vaccination (range: 11.5-82.5%). The possibility of peripheral blood contaminants was excluded due to the absence of CD14+ myeloid cells in aspirate samples. Taken together, the data indicates that GC B cells continue to mature over a period of at least 12-weeks following booster vaccination and raises interesting questions regarding the potential impact on GC reactions of further boosters during this time (see **Table 3**).

**Table 3:** Examples of opportunities to leverage lymph node and bone marrow aspirate sampling to address outstanding vaccinology research questions.

- **The CD19^-^CD38^+^CD138^+^ population** What are the peripheral correlates of vaccine-specific bone marrow CD19^-^CD38^+^CD138^+^ LLPCs? Does this population develop from other CD19^-/+^ bone marrow plasma cell populations? What vaccine delivery parameters are associated with an increase in these LLPCs or precursors? Is there significant variation between antigens? When does this population first appear in the bone marrow following (booster) vaccination?
- **GC duration** What vaccine parameters have the greatest impact on longevity of the GC reaction? How long do GCs last in the presence/absence of subsequent boosters? Can analyses of GC persistence following prime/booster vaccination guide selection of optimal vaccination regimens? Does GC longevity correlate with later serum IgG concentration and/or LLPC seeding in the bone marrow? How do Tfh cell subsets change throughout the GC reaction? Is vaccine antigen detectable throughout?
- **Biomarkers** Are there threshold frequencies of vaccine-specific GC B cells or bone marrow LLPCs that predict durable maintenance of serum antibody? Does this threshold vary by both pathogen and vaccine parameters (e.g. antigen valency)?
- **Clonotype analyses** Do LLPC BCR repertoires have greater somatic hypermutation as compared to circulating vaccine-specific B cell populations? Do monoclonal antibodies generated from these sequences have increased neutralising activity? Can vaccine-specific clones be tracked from draining lymph node GC, to peripheral blood, to the bone marrow? Which memory B cell populations re-enter GCs during secondary exposure? How does the breadth of the BCR repertoire relate to GC kinetics? Are the factors influencing breadth of the humoral response uncoupled from those determining serum antibody durability?
- **Vaccinee confounders** What is the impact of previous or concurrent infections on GC kinetics or LLPC seeding in the bone marrow? Is there a role for vaccinee age or sex in determining the duration and output of GC reactions?
- **Preserving sample integrity** What is the impact of cryopreservation on lymph node and bone marrow aspirate samples with respect to recovery of vaccine-specific populations? Are there differential impacts across immune cell populations? In particular, is plasma cell loss exaggerated as compared to other leucocytes? What are the best practices for excluding the possibility of peripheral blood contaminants in lymph node or bone marrow aspirate samples?

“Vaccine parameters” may include delivery platform (e.g., viral vector, nanoparticle/virus-like particle [VLP], protein, mRNA), adjuvant, antigen characteristic (e.g. size, valency/presence of repeat epitopes), dosing schedule, and ipsilateral versus contralateral boosting.

This FNA work is supported by a recent LN biopsy study in the context of SARS-CoV-2 mRNA vaccine trials (mRNA-1273 or BNT162b2) which showed antigen was detectable in draining lymph node GCs > 8-weeks after booster vaccination [Day 60 (28)]. The authors also report greater follicular hyperplasia and breadth of the antibody response in mRNA vaccinees as compared to naturally infected volunteers, suggestive of a link between lymph node activity and capacity to generate strain-transcendent antibody binding. If so, it is likely that lymph node activity is similarly improved with other SARS-CoV-2 vaccines/vaccine platforms (ChAdOx1-S, Gam-COVID-Vac, BBIBP-CorV) that have likewise induced improved IgG binding to viral variants as compared to infection.

## Bone marrow

Very few human vaccine studies have yet to include bone marrow aspirate sampling – more invasive than lymph node aspirate sampling – and provide direct evidence of vaccine-specific LLPC generation (4, 7, 9, 14). These reports thus provide unique insight into the capacity of various vaccine antigens to induce LLPCs which sustain humoral immunity over time through continued secretion of antibody. Difficulties in recapitulating the bone marrow (LLPC) microenvironment have rendered *in vitro* alternatives uncommon, with the exception of a promising bone marrow mimic model from Lee and colleagues (34, 35). Here, peripheral plasma cells are cultured with a “secretome” from cultured bone marrow mesenchymal stromal cells in the presence of APRIL to model plasma cell longevity in the bone marrow. This assay has yet to be widely adopted, but inclusion in clinical trial immunology alongside direct detection of bone marrow LLPCs may represent a future opportunity to define LLPC biomarkers by *in vitro* assays with peripheral blood, removing the need for bone marrow sampling.

To collect bone marrow aspirate samples, the main parameters to define are site and volume of aspirate. In the human studies highlighted below, a 30ml aspirate from the iliac crest was most commonly reported. There are some indications both that while the iliac crest may be the most practical for human studies, mononuclear cells yields may vary by anatomical site (36), and also that yield/ml may vary by syringe volume (37). Aspirate samples were almost exclusively used for ELISPOT assays, preceded by a plasma cell enrichment step with CD138^+^ magnetic beads (4, 7, 9, 14). Depending on mononuclear cell yields (**Table 4**), IgG alone or IgG/IgM/IgA responses were evaluated. ELISPOTs with matched peripheral blood samples – or probe-based flow cytometry analyses – may also be used at the same time point to ensure vaccine-specific populations detected are not artefacts from peripheral blood contaminants during sampling (9). Other authors have similarly excluded contamination through comparison of CD4+ T cell phenotypes between peripheral blood and bone marrow aspirate samples (7).

**Table 4:** Average cell yields from healthy adult bone marrow aspirates. Site of aspirate and cell yields not reported in (6, 7).

**Vaccination study?**	**Publication**	**Site of aspirate**	**Volume of aspirate (ml)**	**Cell yield per aspirate (average)**	**Cell yield per aspirate (range)**
Historical vaccination; observational study	Mei et al., 2015. *Blood* (4)	Femoral head or sternum or iliac crest*	Not reported		
	Halliley et al., 2015. *Immunity* (5)	Iliac crest	Not reported	1 x 10^4^ BM plasma cells	
Yes	Davis et al., 2020. *Science* (9)	Iliac crest	30	1 x 10^5^ BM plasma cells	6 x 10^4^ – 5 x 10^5^ BM plasma cells
Yes	Kim et al., 2022. *Nature* (14)	Iliac crest	30	Not reported	
No	Turner et al., 2021. *Nature* (38)	Iliac crest	30	Not reported	

*Bone marrow samples aspirated during scheduled hip replacement (femoral head), cardiac surgery (sternum), or treatment for rheumatoid arthritis (iliac crest).

A seminal observational study published in 2015 by Mei and colleagues provided the first description of vaccine-specific B cells in human bone marrow (4). Here, bone marrow was accessible from patients undergoing surgery for other purposes and available for in-depth analysis of plasma cell subsets. The authors detail a novel population of CD19- plasma cells that are enriched in the bone marrow as compared to other compartments, and possess a mature, pro-survival phenotype. Tetanus-specific cells were detected by ELISPOT within both IgG^+^CD19^-^ and IgG^+^CD19^+^ bone marrow plasma cells (*n*=4; range: 0.27-1.56%), but the authors propose the CD19^-^ subset as the true LLPC population. This is due to lower expression of Ki67, higher viability after 3-9 days of *in vitro* culture, and lower IgV_H_ mutation frequencies as compared to blood or bone marrow CD19+ populations. However, these comparisons to other tissues were not from matched volunteers and time since vaccination for the bone marrow volunteers was not known. Regardless, these findings give intriguing insight into the diversity of vaccine-specific plasma cell populations that may reside in the bone marrow.

In the same year, Halliley and colleagues also described a CD19^-^ LLPC bone marrow population, in a study which included analysis of antigen-specific bone marrow plasma cell populations to historical vaccination or infection (5). Here, the authors sorted plasma cells into 4 subsets based on CD19/CD38/CD138 expression from healthy adults and performed ELISPOTs to detect vaccine antigen-specific plasma cells for measles, mumps, seasonal influenza, or tetanus toxoid. Tetanus-specific responses were observed in 4/7 subjects in the CD19^-^CD38^+^CD138^+^ subset with a mean frequency of 0.51% of total IgG antibody-secreting cells (range: 0-1.8%). To confirm the longevity of this CD19^-^CD38^+^CD138^+^ plasma cell population, the authors also looked for measles- and mumps-specific plasma cells in healthy adults with high serum antibody but no history of MMR vaccination (i.e. exposure from childhood measles/mumps/rubella infection over four decades ago). In 10/11 volunteers, antigen-specific plasma cells were detected and again found almost exclusively in the CD19-CD38^+^CD138^+^ subset (mean: 0.6%; range: 0-1.8%). In contrast, responses to a more recent antigen exposure (seasonal influenza vaccination) within the last 1-11 months yielded antigen-specific plasma cells across multiple plasma cell subsets. This led the authors to conclude that short-lived plasma cells may be found within multiple compartments (most convincingly CD19^+^CD38^+^CD138^+^ and CD19^-^CD38^+^CD138^+^), whereas LLPCs are restricted to CD19-CD38^+^CD138^+^. Finally, with a single 64-year old volunteer, the authors isolated and sequenced serum measles- and mumps-specific IgG and compared these sequences to bone marrow plasma cell heavy chain complementarity-determining region 3 (HCDR3) sequences using SEQUEST. Unique measles and mumps serum IgG sequences were matched only with those from the CD19^-^CD38^+^CD138^+^ plasma cells, indicating serum IgG is maintained by plasma cells in this population alone. Elsewhere in the paper, the authors characterise each of these bone marrow plasma cell populations with both flow cytometry and gene expression analyses. This study remains one of the most detailed characterisations of bone marrow plasma cell heterogeneity and underscores the importance of distinguishing between CD19^-/+^ populations.

Further investigation by Groves and colleagues also detected differences in frequencies of vaccine-specific responses within CD19-defined subsets by ELISPOT (6). Here, the authors assayed for responses against influenza, polio, or diphtheria/tetanus/pertussis (Daptacel vaccine). A significantly higher influenza-specific frequency was observed in the CD19- population as compared to CD19^+^ plasma cells (*n*=10), whereas no differences were observed in the Daptacel-specific response. Comparisons for polio were not reported.

Also making use of prior vaccination comes a study from Eberhardt and colleagues who assessed (by ELISPOT) varicella-zoster virus (VZV)-specific plasma cells and CD4^+^ T cells in bone marrow aspirates in 15 adults with a median time since vaccination of 8.6 years (7). The mean frequency of VZV-specific IgG+ plasma cells in the bone marrow was 0.2%, and while VZV-specific memory B cells were detectable in peripheral blood there were no detectable VZV-specific plasma cells in circulation. Conversely, VZV-specific CD4^+^ T cells were identified both in peripheral blood and – at a lower frequency – in the bone marrow by IFN-γ ELISPOT following peptide pool stimulation. The authors did not have statistical power to thoroughly interrogate either the impact of number of doses (1-2) or time since vaccination (range: 0.1-19.6 years) on vaccine-specific LLPC frequency, but the results are highly encouraging for the durability of VZV vaccination.

Building on this work, Davis and colleagues subsequently investigated the induction of bone marrow plasma cells (BMPC) following seasonal influenza vaccine in a large study of 53 adults (9). Significant increases were observed by ELISPOT in influenza-specific IgG cells within the BMPC population from a mean of 0.8% pre-vaccination to 1.9% 4-weeks after vaccination. A subset of volunteers donated a third aspirate 1-year after vaccination, by which stage frequencies of influenza-specific plasma cells had returned to baseline. While the frequencies of influenza-specific IgG at 4-weeks was higher than had been previously observed with VZV or tetanus toxoid (7), this disappearance of the antigen-specific BMPC population by 1-year contributes to our understanding of why vaccine-mediated humoral immunity is not well maintained in the context of influenza. The authors also performed VDJ sequencing with blood ASCs from Day 7 post-vaccination and identified 15 clones that were expanded (>0.5% total ASC sequences). Interestingly, 14 of these clonotypes were also expanded in the BMPC population (*n*=4 vaccinees) indicating that for influenza vaccination the Day 7 ASC population may be a good predictor of subsequent BMPC. The sequencing data also indicated variation in the longevity of each of the clones, and that those that were lost were vaccine-induced rather than pre-existing. The lack of overlap between vaccine-induced and pre-existing clones is consistent with negative feedback mechanism of circulating antibody at the time of vaccination, i.e. binding to cognate epitope and blocking new responses. Further bone marrow studies should include similar VDJ sequencing – ideally with a method to enrich for or isolate vaccine-specific BMPCs – to further interrogate the relationship between circulating and bone marrow B cell populations.

At the time of writing, the most recent foray into bone marrow aspirate sampling in the vaccinology field came from Kim and colleagues with a COVID mRNA vaccine study [also referenced above in the context of lymph node sampling (14)]. Here, bone marrow aspirates were taken from 11 vaccinees 6-months after the booster spike mRNA vaccination. Spike-specific IgG^+^ BMPCs were detected in 9/11 vaccinees with a median frequency of 0.06% within the IgG-secreting BMPCs. This was significantly lower than the median frequencies of influenza-specific (1.4%) or tetanus/diphtheria-specific (0.15%) IgG^+^ BMPCs detected in the same samples. The authors speculate that this is due to the greater number of antigen targets and/or more frequent exposures to influenza/tetanus/diphtheria as compared to SARS-CoV-2; this raises questions about potential requirements for additional booster dosing, antigen inclusion, or adjuvant reformulation for optimisation of SARS-CoV-2 vaccine-mediated protection. It is also unclear why 2/11 vaccinees did not have detectable BMPCs. On a more encouraging note, the spike-specific BMPCs showed increased somatic hypermutation as compared to other plasma cell populations in the blood and lymph node, while hierarchical phylogenetic modelling indicates a close relationship between lymph node and bone marrow plasma cells. This data supports a model of SHM accumulation along the differentiation trajectory from early plasmablasts, to mBC and lymph node GC B or plasma cells, followed by bone marrow plasma cells with the highest SHM. At the late 6-month time points BM LLPC SHM indeed correlated with serum avidity. This work remains the only vaccine study to include paired draining lymph node aspirates and bone marrow aspirates.

Finally, while infection studies are outside the scope of this review, attention must be drawn to a further study by Turner and colleagues which sought to determine whether LLPCs were induced in the context of SARS-CoV-2 infection (38). Here, spike-specific cells were detected within IgG- or IgA-secreting bone marrow plasma cells in 15/18 study participants by ELISPOT 7-months after the onset of mild symptoms (medians: IgG ≈ 0.1%, IgA ≈ 0.02%). A further bone marrow aspirate sample was obtained from 5/19 volunteers after 11-months, where 4/5 retained detectable spike-specific LLPCs. Spike-specific LLPCs correlated modestly with serum anti-spike IgG by Spearman correlation (*n*=18, *r*=0.48, *p*=0.046). Of potential great significance for the design of future studies, the authors also successfully detected spike-specific LLPCs at a comparable frequency by flow cytometry using intracellular staining with fluorophore-conjugated spike probes. High expression of CD38 and negligible expression of the proliferation marker Ki67 were consistent with a stable phenotype. Influenza-specific and tetanus/diphtheria-specific LLPCs were similarly detectable in control and convalescent volunteers by both ELISPOT and (for influenza) flow cytometry. Doubts have previously been raised regarding the feasibility of probe-based detection of antigen-specific plasma cells due to the downregulation of the BCR and this work therefore provides an encouraging proof of concept for this approach.

## Non-human primates

While a comprehensive picture of insights gained from lymphoid tissues sampling in NHPs is outside the scope of this review, it is informative to highlight examples of studies where this approach has provided key insights, such as on the impact of adjuvant selection and dosing schedules, or potentially informative techniques that could be employed in human studies.

Starting with examples of draining lymph node analyses, here we have multiple examples where NHP analyses have contributed to our understanding, particularly with respect to the role of antigen dosing kinetics and adjuvant selection in driving vaccine-specific B cell responses and neutralising antibodies (nAbs). This work has largely been done in the context of HIV development with rhesus macaques. nAb development requires affinity maturation which occurs in GC reactions; draining lymph node analysis can therefore provide crucial insight into the GC Tfh/B cell activation required for this process.

The first study came from Pauthner and colleagues in 2017 with a comparison of 6-week versus 8-week dosing prime/boost vaccination regimen, intramuscular versus subcutaneous vaccination routes, and conventional versus extended immunogen release using an osmotic pump [*n*=6-12 per group (39)]. The authors included FNA sampling of draining lymph nodes at baseline and then 3-weeks after each of the three immunisations. This longitudinal monitoring of the draining lymph nodes allowed the authors to monitor GC responses and establish that total GC B cells tracked with sera nAb. Moreover, the frequency of total GC B cells 3-weeks after the first vaccination correlated with nAb 2-weeks after the second vaccination (r=0.64, *p*=0.026). The longer dosing interval, subcutaneous vaccination, and prolonged immunogen release all improved GC B cell and nAb responses. With respect to the route of vaccination, by using Evans Blue the authors were further able to establish that subcutaneous vaccination induces more rapid “antigen” drainage to the lymph node, consistent with superior induction of GC B and Tfh cell responses. However, it was the prolonged immunogen release that yielded the greatest increase in GC activity and downstream nAb development, though further dose-matched comparisons and an understanding of how well Evans Blue (a small molecule dye) recapitulates formulated vaccine antigen draining is needed. A more recent study has proposed tattoo ink as an alternative for identifying the antigen draining lymph nodes within a lymph node cluster (40).

Indeed, building on this and in the context of efforts to improve neutralising antibody responses following HIV-1 vaccination, Cirelli and colleagues compared Env-specific GC B cell and Tfh cell responses in rhesus macaques following three different antigen delivery strategies [*n*=3-6 per group (41)]. Using longitudinal draining lymph node aspirate sampling – the first kinetic analysis of this type in any species – the authors demonstrated that antigen delivery by an osmotic pump rather than a single bolus vaccination increased peak Env-specific response in the GC approximately 5-fold, which correlated with neutralising responses at a later time point (r=0.673, *p*=0.0008). Of particular interest was the quality and quantity of the GC B cell response: Env-specific GC B cells not only continued to increase between 4-8 weeks after vaccination rather than plateauing as in bolus vaccinees, but showed increased clonal diversity. An escalating dose vaccination regimen (mimicking natural infection) recapitulated these osmotic pump improvements over bolus vaccination. These differences were only detectable due to direct examination of GC populations in draining lymph node aspirates and suggest an impact of dosing regimen on GC longevity.

Silva and colleagues have also investigated the capacity of saponin adjuvants to impact events in draining lymph nodes in rhesus macaques [*n*=6 per group (42)]. Building on the previous study which had employed Quil-A immune-stimulating complexes (ISCOMs) (41), the authors moved to a saponin/MPLA adjuvant (SMNP) which had shown improved effectiveness in mice with indications of increased lymphatic transport of antigen to GC B cells (42). Here, they observed robust Env-specific GC responses in lateral and central axillary lymph nodes near the immunisation site. In comparison to the two other adjuvants tested – cyclic dinucleotides (CDNs) and CpG – frequencies of Env-specific B cells within GC B cells were higher in SMNP-vaccinated macaques six weeks after a single vaccination (*p*=0.0031; medians [range]: SMNP 6.86% [4.50-12.40%], CDN 3.77% [0.90-7.35%], CpG 2.33% [1.93-2.49%]). Total Tfh cells (CXCR5^hi^PD1^hi^) and GC B cells (IgG^+^Ki67^+^Bcl6^+^) were also significantly higher at this time point. The authors concluded that SMNP is a potent adjuvant for humoral immunity in NHPs and therefore promising for clinical work alongside the extended delivery mechanisms described above. A direct comparison of GC longevity for the same antigen with different vaccine adjuvants or platforms (including protein) remains to be confirmed in human studies.

Finally, a study by Kelly and colleagues draws attention to the importance of understanding inconsistencies in draining lymph node GC kinetics between small and large animal models (43). Here, the authors explored the mechanisms underpinning superior humoral immunogenicity and protective efficacy of an influenza nanoparticle vaccine platform as compared to a soluble HA protein vaccination. While in mice the nanoparticle induced higher antibody responses, expanded and persistent GC reactions in draining lymph nodes, no such differences in immunogenicity were observed between platforms in a macaque model, suggesting a different impact on nanoparticle formulation on GC kinetics and output. This works underlies the importance of using relevant preclinical models to interrogate lymphoid tissue vaccinology if sampling is not feasible in clinical trials. The authors did note that HA-specific GC B cells were detectable by probe staining in 3/5 nanoparticle-vaccinated macaques as compared to 0/5 soluble protein-vaccinated macaques, but no statistical comparisons were reported for antigen-specific GC responses.

With respect to bone marrow analyses, Hammarlund and colleagues investigated the longevity of rhesus bone marrow plasma cells in the absence of memory B cells or other CD20^+^ B cell populations following surgical removal of spleen, lymph nodes, and CD20^+^ cell depletion with Rituximab(*n*=2-4 per group (36). The authors detected tetanus-specific bone marrow plasma cells 10 years after vaccination with an acellular diphtheria/tetanus/pertussis (DtaP) vaccine by ELISPOT, largely in the CD20^-^CD38^+^ compartment. The rhesus macaques had also received BrdU for 12 days after the 2^nd^/3^rd^/4^th^ vaccinations. By detecting BrdU^+^ plasma cells in the bone marrow 10 years after vaccination and BrdU administration, the authors were also able to infer that LLPCs persist for a long time in the absence of cell division. Similar studies in humans using similar labelling approaches, for example with deuterated water, have not yet been reported but could represent an informative approach to determining half-lives of vaccine-specific LLPCs. Also of note was the uneven distribution of vaccine-specific plasma cells across bone marrow sites. The reason for this is unknown and, again, understanding the analogous distribution homing patterns in humans will be important.

Recent work by Kasturi and colleagues demonstrated that protection with an RBD-based vaccine against SARS-CoV-2 challenge was improved with use of the 3M-052-alum adjuvant, as opposed to RBD formulated with alum alone [*n*=4-5 per group (44)]. However, important insight into the potential duration of antibody-mediated protection was provided by serial bone marrow aspirate sampling. While a significantly higher frequency of RBD-specific IgG^+^ and IgA^+^ ASCs within the bone marrow mononuclear cell (BMMNC) population was detected 2-weeks after the final (third) vaccination with 3M-052-alum (IgG^+^ mean ≈15 per million BMMNC) versus alum alone (IgG^+^ mean ≈3 per million BMMNC), this difference was no longer present at 4-weeks post final vaccination. By this point, the means were <5 in both groups for both isotypes. The reason for this limited induction and maintenance of LLPCs in the bone marrow is not clear, but suggests relatively short life span or poor quality LLPC differentiation. This insight would not have been revealed in the absence of bone marrow sampling as the final time point of antibody analyses was also week 13, at which point titres were still maintained near peak levels (44).

These findings are in contrast to a previous 3M-052-alum study with the HIV-1 antigen Env, where vaccination drove superior LLPC seeding as compared to alum alone (45). Here, after a third dose, the mean Env-specific IgG^+^ ASCs per million BMMNC was >200 in the 3M-052-alum group and comparable to the RBD study with alum alone (<10 per million BMMNC). A late time point at week 65 (approximately 25 weeks after the final fourth vaccination which did not significantly boost above third dose) showed promising maintenance of these Env-specific IgG^+^ LLPCs with a median >100 per million BMMNCs, 10-fold higher than in alum-only NHPs. These two studies highlight that while the impact of adjuvant selection may be consistent between antigen, there may also be substantial variation attributable to the antigen. Such differences may only be revealed by bone marrow aspirate sampling in an early phase trial. Further immunokinetic work would be facilitated by larger group sizes to improve statistical power, particularly when variable assays such as ELISPOTs are employed (*n*=4-6 per group in work described above), and statistical comparisons between time points within a group to formally compare cellular half-lives. Whether these same kinetics translate to human trials remains to be established, but this comparison will likely be feasible soon as 3M-052-alum continues to be under clinical development.

## Discussion

Human vaccination studies incorporating lymph node and bone marrow aspirate sampling have made crucial contributions to our understanding of the Tfh and B cell drivers of humoral immunity, with the majority of findings reported within the last few years. Meanwhile, exciting data from NHP studies should prompt greater creativity with vaccine dosing regimens to maximise GC responses (39, 41). Indeed, there remains enormous potential of lymphoid tissue sampling to reveal further critical mechanistic insights into vaccine immunogenicity and efficacy (**Table 3**).

So why are these sampling approaches not more widely deployed? One of the main barriers to lymphoid tissue sampling as compared to venous blood draws continues to be the specialist personnel required: a radiologist/ultrasound technician for lymph nodes and a haematologist for bone marrow, versus more widely accessible phlebotomists for peripheral blood. For bone marrow aspirates, volunteer discomfort is also substantially greater than during a venous blood draw. It is therefore important that, for a given study, the data sought from lymphoid samples is not available from peripheral blood samples alone. The publications detailed above provide many examples of where this is the case, but further emphasis on key themes here is warranted.

First, of particular note are studies focused on understanding the longevity of humoral immunity, a perennial issue for vaccinologists and of utmost importance for the public health impact of a given vaccine. The population of B cells mediating durability of humoral immunity are the LLPCs in the bone marrow. While the work highlighted above shows that these vaccine-specific LLPCs are detectable by ELISPOT and flow cytometry for many months or years after exposure (5, 7, 14), circulating vaccine-specific memory B cells wane below the limit of detection of these current techniques. A worthy longer-term goal would be to establish peripheral blood correlates of the bone marrow populations of interest e.g. LLPC precursors (**Table 3**). As emphasised by Halliley and colleagues, these analyses must take into account the heterogeneity of bone marrow plasma cell populations: LLPCs are most likely contained solely within the CD19^-^CD38^+^CD138^+^ compartment, while other bone marrow plasma cell populations may be more transient (5). This work will therefore likely require moving beyond ELISPOTs towards more refined single-cell analyses to interrogate bone marrow populations. Incorporating probe-based methods to detect and isolate antigen-specific cells will likely form a key component of this future work and open doors to more nuanced downstream flow cytometry and single-cell RNA sequencing analyses (16, 38). For example, as observed by Davis and colleagues, for bone marrow plasma cells to fully differentiate into LLPCs they need to not only reach the appropriate survival niche and compete for space, but also undergo gene expression and metabolic changes to promote longevity (9). If a vaccine-specific LLPC population is not established, this suggests one of these processes is not occurring sufficiently. Identifying the problem will likely rely on single cell studies to identify which subsets are successfully differentiating, and linking these to LLPC-precursor clones detectable in the blood during expansion and homing to the bone marrow.

Equally, many of the lymph node analyses highlighted above report findings that would not have been observed through peripheral blood sampling alone. In particular, there is clearly notable value in lymph node aspirate sampling for directly comparing magnitude and longevity of GC responses between vaccine antigens, adjuvants, platforms, and dosing regimens. Longer GC reactions are almost certainly beneficial and likely increase LLPC output, as well as drive greater affinity maturation to improve serum IgG avidity and likelihood of bnAb development. The persistence of GCs may also be of great relevance for optimising booster dosing intervals.

With respect to BCR repertoire, paired analyses of peripheral memory B cells (pre-booster) and GC B cells (post-booster) will likely yield insights into the relationship between primary and secondary responses. Preclinical studies have shown that memory B cell populations vary in their capacity to re-enter GCs during subsequent exposures (46, 47) but limited comparable work has been done in humans (**Table 4**). In scenarios where post-vaccination serum IgG responses have high peaks but are poorly maintained, it would be highly useful to determine whether the underlying mechanism relates to poor recruitment of memory B cells back to GCs. Likewise, there is an opportunity to link LLPC clones to these earlier activation events. Understanding these clonal dynamics may thus have a profound impact of vaccination strategies.

To conclude, the burgeoning field of lymphoid tissue vaccinology holds exciting opportunities to understand the cellular interactions and populations underpinning humoral immunity. While existing data is dominated by work in the SARS-CoV-2 field, the sampling and analysis approaches are translatable to a wider range of vaccine candidates and pathogens.

## Author contributions

All authors listed have made a substantial, direct, and intellectual contribution to the work and approved it for publication.

## Funding

CN was supported by a Sir Henry Wellcome Postdoctoral Fellowship (209200/Z/17/Z). The authors have no financial conflicts of interest to report.

## Conflict of interest

The authors declare that the research was conducted in the absence of any commercial or financial relationships that could be construed as a potential conflict of interest.

## Publisher’s note

All claims expressed in this article are solely those of the authors and do not necessarily represent those of their affiliated organizations, or those of the publisher, the editors and the reviewers. Any product that may be evaluated in this article, or claim that may be made by its manufacturer, is not guaranteed or endorsed by the publisher.

## References

[1] Amanna IJ Carlson NE Slifka MK . Duration of humoral immunity to common viral and vaccine antigens. N Engl J Med (2007) 357:1903–15. doi: 10.1056/NEJMoa066092 17989383

[2] Slifka MK Amanna IJ . Role of multivalency and antigenic threshold in generating protective antibody responses. Front Immunol (2019) 10:956. doi: 10.3389/fimmu.2019.00956 31118935 PMC6504826

[3] Lau D Lan LY Andrews SF Henry C Rojas KT Neu KE . Low CD21 expression defines a population of recent germinal center graduates primed for plasma cell differentiation. Sci Immunol (2017) 2(7):eaai8153. doi: 10.1126/sciimmunol.aai8153 28783670 PMC5896567

[4] Mei HE Wirries I Frolich D Brisslert M Giesecke C Grun JR . A unique population of IgG-expressing plasma cells lacking CD19 is enriched in human bone marrow. Blood (2015) 125:1739–48. doi: 10.1182/blood-2014-02-555169 25573986

[5] Halliley JL Tipton CM Liesveld J Rosenberg AF Darce J Gregoretti IV . Long-lived plasma cells are contained within the CD19(-)CD38(hi)CD138(+) subset in human bone marrow. Immunity (2015) 43:132–45. doi: 10.1016/j.immuni.2015.06.016 PMC4680845 26187412

[6] Groves CJ Carrell J Grady R Rajan B Morehouse CA Halpin R . CD19-positive antibody-secreting cells provide immune memory. Blood Adv (2018) 2:3163–76. doi: 10.1182/bloodadvances.2017015172 PMC6258909 30478153

[7] Eberhardt CS Wieland A Nasti TH Grifoni A Wilson E Schmid DS . Persistence of varicella-zoster virus-specific plasma cells in adult human bone marrow following childhood vaccination. J Virol (2020) 94(13):e02127-19. doi: 10.1128/JVI.02127-19 32321817 PMC7307153

[8] Turner JS Zhou JQ Han J Schmitz AJ Rizk AA Alsoussi WB . Human germinal centres engage memory and naive b cells after influenza vaccination. Nature (2020) 586:127–32. doi: 10.1038/s41586-020-2711-0 PMC7566073 32866963

[9] Davis CW Jackson KJL McCausland MM Darce J Chang C Linderman SL . Influenza vaccine-induced human bone marrow plasma cells decline within a year after vaccination. Science (2020) 370(6513):237–41. doi: 10.1126/science.aaz8432 PMC10155619 32792465

[10] Turner JS O’Halloran JA Kalaidina E Kim W Schmitz AJ Zhou JQ . SARS-CoV-2 mRNA vaccines induce persistent human germinal centre responses. Nature (2021) 596:109–13. doi: 10.1038/s41586-021-03738-2 PMC8935394 34182569

[11] Mudd PA Minervina AA Pogorelyy MV Turner JS Kim W Kalaidina E . SARS-CoV-2 mRNA vaccination elicits a robust and persistent T follicular helper cell response in humans. Cell (2022) 185:603–613 e15. doi: 10.1016/j.cell.2021.12.026 35026152 PMC8695127

[12] Law H Mach M Howe A Obeid S Milner B Carey C . Early expansion of CD38+ICOS+ GC tfh in draining lymph nodes during influenza vaccination immune response. iScience (2022) 25:103656. doi: 10.1016/j.isci.2021.103656 35028536 PMC8741621

[13] Lederer K Bettini E Parvathaneni K Painter MM Agarwal D Lundgreen KA . Germinal center responses to SARS-CoV-2 mRNA vaccines in healthy and immunocompromised individuals. Cell (2022) 185:1008–1024 e15. doi: 10.1016/j.cell.2022.01.027 35202565 PMC8808747

[14] Kim W Zhou JQ Horvath SC Schmitz AJ Sturtz AJ Lei T . Germinal centre-driven maturation of b cell response to mRNA vaccination. Nature (2022) 604:141–5. doi: 10.1038/s41586-022-04527-1 PMC9204750 35168246

[15] Noe A Cargill TN Nielsen CM Russell AJC Barnes E . The application of single-cell RNA sequencing in vaccinology. J Immunol Res (2020) 2020:8624963. doi: 10.1155/2020/8624963 32802896 PMC7411487

[16] Utset HA Guthmiller JJ Wilson PC . Bridging the b cell gap: Novel technologies to study antigen-specific human b cell responses. Vaccines (Basel) (2021) 9(7):711. doi: 10.3390/vaccines9070711 34358128 PMC8310089

[17] Broketa M Bruhns P . Single-cell technologies for the study of antibody-secreting cells. Front Immunol (2021) 12:821729. doi: 10.3389/fimmu.2021.821729 35173713 PMC8841722

[18] Woodruff MC Nguyen DC Faliti CE Saini AS Lee FE Sanz I . Response under pressure: deploying emerging technologies to understand b-cell-mediated immunity in COVID-19. Nat Methods (2022) 19:387–91. doi: 10.1038/s41592-022-01450-1 PMC9703369 35396475

[19] Tan HX Wragg KM Kelly HG Esterbauer R Dixon BJ Lau JSY . Cutting edge: SARS-CoV-2 infection induces robust germinal center activity in the human tonsil. J Immunol (2022) 208:2267–71. doi: 10.4049/jimmunol.2101199 35487578

[20] Amodio D Cotugno N Macchiarulo G Rocca S Dimopoulos Y Castrucci MR . Quantitative multiplexed imaging analysis reveals a strong association between immunogen-specific b cell responses and tonsillar germinal center immune dynamics in children after influenza vaccination. J Immunol (2018) 200:538–50. doi: 10.4049/jimmunol.1701312 PMC5760299 29237774

[21] Landsverk OJ Snir O Casado RB Richter L Mold JE Reu P . Antibody-secreting plasma cells persist for decades in human intestine. J Exp Med (2017) 214:309–17. doi: 10.1084/jem.20161590 PMC5294861 28104812

[22] Wagar LE Salahudeen A Constantz CM Wendel BS Lyons MM Mallajosyula V . Modeling human adaptive immune responses with tonsil organoids. Nat Med (2021) 27:125–35. doi: 10.1038/s41591-020-01145-0 PMC7891554 33432170

[23] Goyal G Prabhala P Mahajan G Bausk B Gilboa T Xie L . Ectopic lymphoid follicle formation and human seasonal influenza vaccination responses recapitulated in an organ-on-a-Chip. Adv Sci (Weinh) (2022) 9:e2103241. doi: 10.1002/advs.202103241 35289122 PMC9109055

[24] Walker EB Miller W Haley D Floyd K Curti B Urba WJ . Characterization of the class I-restricted gp100 melanoma peptide-stimulated primary immune response in tumor-free vaccine-draining lymph nodes and peripheral blood. Clin Cancer Res (2009) 15:2541–51. doi: 10.1158/1078-0432.CCR-08-2806 19318471

[25] Slingluff CL Jr. Petroni GR Yamshchikov GV Barnd DL Eastham S Galavotti H . Clinical and immunologic results of a randomized phase II trial of vaccination using four melanoma peptides either administered in granulocyte-macrophage colony-stimulating factor in adjuvant or pulsed on dendritic cells. J Clin Oncol (2003) 21:4016–26. doi: 10.1200/JCO.2003.10.005 14581425

[26] Patricia D’Souza M Allen MA Baumblatt JAG Boggiano C Crotty S Grady C . Innovative approaches to track lymph node germinal center responses to evaluate development of broadly neutralizing antibodies in human HIV vaccine trials. Vaccine (2018) 36:5671–7. doi: 10.1016/j.vaccine.2018.07.071 30097219

[27] Havenar-Daughton C Carnathan DG Torrents de la Pena A Pauthner M Briney B Reiss SM . Direct probing of germinal center responses reveals immunological features and bottlenecks for neutralizing antibody responses to HIV env trimer. Cell Rep (2016) 17:2195–209. doi: 10.1016/j.celrep.2016.10.085 PMC5142765 27880897

[28] Roltgen K Nielsen SCA Silva O Younes SF Zaslavsky M Costales C . Immune imprinting, breadth of variant recognition, and germinal center response in human SARS-CoV-2 infection and vaccination. Cell (2022) 185:1025–1040 e14. doi: 10.1016/j.cell.2022.01.018 35148837 PMC8786601

[29] Ben-Othman R Cai B Liu AC Varankovich N He D Blimkie TM . Systems biology methods applied to blood and tissue for a comprehensive analysis of immune response to hepatitis b vaccine in adults. Front Immunol (2020) 11:580373. doi: 10.3389/fimmu.2020.580373 33250895 PMC7672042

[30] Tatovic D Young P Kochba E Levin Y Wong FS Dayan CM . Fine-needle aspiration biopsy of the lymph node: A novel tool for the monitoring of immune responses after skin antigen delivery. J Immunol (2015) 195:386–92. doi: 10.4049/jimmunol.1500364 26026065

[31] Yang JHM Khatri L Mickunas M Williams E Tatovic D Alhadj Ali M . Phenotypic analysis of human lymph nodes in subjects with new-onset type 1 diabetes and healthy individuals by flow cytometry. Front Immunol (2019) 10:2547. doi: 10.3389/fimmu.2019.02547 31749806 PMC6842967

[32] Havenar-Daughton C Newton IG Zare SY Reiss SM Schwan B Suh MJ . Normal human lymph node T follicular helper cells and germinal center b cells accessed *via* fine needle aspirations. J Immunol Methods (2020) 479:112746. doi: 10.1016/j.jim.2020.112746 31958451 PMC7200018

[33] Lindgren G Ols S Thompson EA Lore K . Comparative analysis of the germinal center response by flow cytometry and immunohistology. J Immunol Methods (2019) 472:16–24. doi: 10.1016/j.jim.2019.06.010 31194971

[34] Nguyen DC Garimalla S Xiao H Kyu S Albizua I Galipeau J . Factors of the bone marrow microniche that support human plasma cell survival and immunoglobulin secretion. Nat Commun (2018) 9:3698. doi: 10.1038/s41467-018-05853-7 30209264 PMC6135805

[35] Garimalla S Nguyen DC Halliley JL Tipton C Rosenberg AF Fucile CF . Differential transcriptome and development of human peripheral plasma cell subsets. JCI Insight (2019) 4(9):e126732. doi: 10.1172/jci.insight.126732 31045577 PMC6538338

[36] Hammarlund E Thomas A Amanna IJ Holden LA Slayden OD Park B . Plasma cell survival in the absence of b cell memory. Nat Commun (2017) 8:1781. doi: 10.1038/s41467-017-01901-w 29176567 PMC5701209

[37] Hernigou P Homma Y Flouzat Lachaniette CH Poignard A Allain J Chevallier N . Benefits of small volume and small syringe for bone marrow aspirations of mesenchymal stem cells. Int Orthop (2013) 37:2279–87. doi: 10.1007/s00264-013-2017-z PMC3824897 23881064

[38] Turner JS Kim W Kalaidina E Goss CW Rauseo AM Schmitz AJ . SARS-CoV-2 infection induces long-lived bone marrow plasma cells in humans. Nature (2021) 595:421–5. doi: 10.1038/s41586-021-03647-4 34030176

[39] Pauthner M Havenar-Daughton C Sok D Nkolola JP Bastidas R Boopathy AV . Elicitation of robust tier 2 neutralizing antibody responses in nonhuman primates by HIV envelope trimer immunization using optimized approaches. Immunity (2017) 46:1073–1088 e6. doi: 10.1016/j.immuni.2017.05.007 28636956 PMC5483234

[40] Barber-Axthelm IM Kelly HG Esterbauer R Wragg KM Gibbon AM Lee WS . Coformulation with tattoo ink for immunological assessment of vaccine immunogenicity in the draining lymph node. J Immunol (2021) 207:735–44. doi: 10.4049/jimmunol.2001299 34244296

[41] Cirelli KM Carnathan DG Nogal B Martin JT Rodriguez OL Upadhyay AA . Slow delivery immunization enhances HIV neutralizing antibody and germinal center responses *via* modulation of immunodominance. Cell (2019) 177(5):1153–71.e28. 31080066 10.1016/j.cell.2019.04.012 PMC6619430

[42] Silva M Kato Y Melo MB Phung I Freeman BL Li Z . A particulate saponin/TLR agonist vaccine adjuvant alters lymph flow and modulates adaptive immunity. Sci Immunol (2021) 6(66):eabf1152. 34860581 10.1126/sciimmunol.abf1152 PMC8763571

[43] Kelly HG Tan HX Juno JA Esterbauer R Ju Y Jiang W . Self-assembling influenza nanoparticle vaccines drive extended germinal center activity and memory b cell maturation. JCI Insight (2020) 5(10):e136653. doi: 10.1172/jci.insight.136653 32434990 PMC7259527

[44] Pino M Abid T Pereira Ribeiro S Edara VV Floyd K Smith JC . A yeast expressed RBD-based SARS-CoV-2 vaccine formulated with 3M-052-alum adjuvant promotes protective efficacy in non-human primates. Sci Immunol (2021) 6(61):eabh3634. doi: 10.1126/sciimmunol.abh3634 34266981 PMC9119307

[45] Kasturi SP Rasheed MAU Havenar-Daughton C Pham M Legere T Sher ZJ . 3M-052, a synthetic TLR-7/8 agonist, induces durable HIV-1 envelope-specific plasma cells and humoral immunity in nonhuman primates. Sci Immunol (2020) 5(48):eabb1025. 32561559 10.1126/sciimmunol.abb1025 PMC8109745

[46] Laidlaw BJ Ellebedy AH . The germinal centre b cell response to SARS-CoV-2. Nat Rev Immunol (2022) 22:7–18. doi: 10.1038/s41577-021-00657-1 34873279 PMC8647067

[47] Zuccarino-Catania GV Sadanand S Weisel FJ Tomayko MM Meng H Kleinstein SH . CD80 and PD-L2 define functionally distinct memory b cell subsets that are independent of antibody isotype. Nat Immunol (2014) 15:631–7. doi: 10.1038/ni.2914 PMC4105703 24880458

